# Supplementary material for: A comparison of anatomic and cellular transcriptome structures across 40 human brain diseases
Source: PLoS Biol. 2023 Apr 20;21(4):e3002058. doi: 10.1371/journal.pbio.3002058 (PMC10118126; doi:10.1371/journal.pbio.3002058)
Supplement: Attachment — Submitted filename: PLOS_BIOLOGY_review_response_letter.pdf [file pbio.3002058.s010.pdf]

Please find enclosed the revised version of our paper (PBIOLOGY-D-22-00725) titled “Anatomic and cellular transcriptome structure of human brain disease”. We thank the editor and reviewers for their constructive feedback. We have addressed the points that were raised by the reviewers; these suggestions and comments have greatly helped in strengthening the manuscript. Below is our response, highlighted in color, to these issues. We have significantly rewritten the manuscript to address the points made by the reviewers. In addition, we have marked the newly added sections throughout the text. Please Note that these are the sections that are either completely new or completely changed and not the only changes in the manuscript, since a large portion of the text has been revised and rewritten for clarity we didn’t highlight all of them.

Reviewer #1: Zeighami et al. leveraged rich data resources (both neuroanatomical and single cell transcriptomes) from the Allen brain atlas to investigate the expression of disease-associated genes in adult human and mouse brain.The authors concluded that disease risk genes of different brain disorders exhibit different anatomic transcriptomic signatures. By analysis of single cell transcriptomes, they found cell type-dependent gradients that separate neurodegenerative, psychiatric, and substance abuse disorders.

We would like to reiterate the reviewer's summary to emphasize that the overarching goal of the study was to investigate and describe the large-scale anatomic and cell type patterning of disease risk associated genes in the brain. Using anatomically mapped tissue sources and cell types, we observe that disease risk genes show convergent physiological based expression patterns that associate diseases in expected and sometimes less expected ways. While this approach is at a lower resolution that is required to derive detailed translational interpretation, a main novelty we believe is precisely in describing and summarizing the brain-wide transcriptomic architecture of genetic risk for major classes of brain disease. We compared analysis throughout the work to phenotypic classification based on the highly cited Global Burden of Disease (GBD) study from the (Institute for Health Metrics (IHME), healthdata.org). This is an important bridging link between epidemiological classification and transcriptomics which should be of interest to scientists in a large community. Whereas the molecular basis of disease will ultimately reveal deeper associations which may lead to therapeutic options, we argue that the present approach is a step toward a computationally driven approach that will use transcriptomic and cell/pathway data to inform brain disorder classification. While cell type specific data will provide the deepest and most informative resolution required to establish the molecular basis of disease risk, tissue based anatomic sampling provides a logical approach for comparative analysis across major brain patterning and forms a natural way to describe overall structure. In addition to having added new analyses, we have substantially rewritten the manuscript to better highlight the findings and significance, and we believe, clarified the presentation.

R1-1: The success of the authors' strategy hinges on gene selection for each of these complex and very different brain disorders. Each disease-associated gene carries equal weight in the analysis. This is potentially problematic because 1) the disease burden carried by each gene can vary significantly (some carry significant burden whereas others are risk factors), 2) the strength of the evidence supporting each gene also varies a great deal (some are convergently supported

by multiple large cohort studies whereas others have conflicting data), 3) the nature of the mutations causing each disease (i.e. loss-of-function, gain-of-function, neomorphic, regulatory) or the mode of inheritance were also not considered.

For genes associated with autism spectrum disorder, some carry a large effect in a nearly Mendelian way (e.g. CHD8), whereas others (e.g. MTHFR) carry weak and controversial associations. As far as I can tell, these genes carry equal weight in the authors' analysis.For genes associated with William syndrome, which results from copy number loss at 7q11, there is an additional issue. It is thought that only some of the genes within the CNV interval contribute to William syndrome phenotypes. It is therefore likely that some of the genes selected by the authors for analysis contribute to little or none of the disease (they merely fall within the CNV interval).

We thank the reviewer for making these extremely important observations. The reviewer correctly observes the complexity of identifying the actual extent of contributions of disease-associated genes and that this may vary significantly between disorders, with varying strength of evidence for distinct genes across multimodal experimental data sources. These sources of variation are subtle, not well elucidated in literature, and it is a major challenge of the translational studies to identify meaningful association and weights. We acknowledge that a fully detailed analysis would require an understanding of how different genetic variants or copy number variations contribute to the disease process, whether the respective mechanism is due to loss of function or gain of function, and whether genes involved are rare or common in the patient population. The approach of DisGeNET prioritization relies on a statistical point of view, across these 40 disorders and 1646 genes, affected brain structure, neural pathway, and cell type that is based on the normative expression profile of each gene. Naturally the utility of this assumption is potentially less meaningful when it comes to the effects of individual genes involved and their contribution to risk.

There is undoubtedly great variation in the magnitude of effect of individual genes and disease risk and any actionable and detailed comparative analysis would need to address this. However, at our present level of understanding of many diseases, it is difficult to ascertain precisely how to quantify this risk. Nonetheless the reviewers critique remains valid indicating more care may be necessary in disease gene risk prioritization. To address these issues, we conducted further analysis to evaluate the effect of gene importance as reflected in the literature. We used the literature based gene disease association weights provided by the DisGeNET dataset where each association has a gene-disease association (GDA) score based on the following formula: **GDA-score = C + M + I + L** where C is based on curated data sources, M is based on mouse and rat animal model reports, I is inferred GDAs from the Human Phenotype Ontology, VDAs reported by Clinvar, the GWAS catalog and GWAS database, and finally L is based on number of publications reporting the given GDA. Using the **GDA-score** for each gene disease association we then calculated a weighted average expression representing the disease related global gene expression pattern across brain regions and cell types as in **Figure 1.** In this new analysis, weighted average replaces the equally weighted average used in the main analysis. We recomputed the main analysis for the HBA dataset with results shown in **Suppl. Fig. 8**. In the new analysis 5 diseases will be removed due to insufficient data (migraine disorders, obsessive-compulsive disorder, frontotemporal lobar degeneration, dementia, heroin dependence.) The new

result illustrates a very similar pattern across brain regions grouping similar disease classes, confirming the original classification presented in **Figure 1** and showing 85% agreement between class assignment of remaining diseases. To present the results with the fewest assumptions, accounting for potential variability of the GDA-score, and retaining the 40 diseases, we hope the reviewer will agree with our decision to retain the original Figure due to similarity of results. Please see supplementary methods for more details.

R1-3: The nature of the disease-causing mutations needs to be considered. Genes can cause disease in many ways. The authors' strategy may work well for loss-of-function mutations but is likely not to work for gain-of-function, neomorphic, or regulatory mutations. The nature or direction of effect of mutations do not seem to have been considered; this is a major caveat of the study.

The reviewer's point is well taken, and this level of mechanism is not accounted for in our study. In this analysis we are not making claims about the mechanism of action, rather we are saying that disease associated genes act in cell types in which they are expressed and that these have characteristic expression patterns in the brain. We admit that we have not provided a global classification of brain diseases accounting for disease-causing mutations, gain or loss of function and related considerations, and such a study would be of paramount interest. We have commented on this limitation in the discussion.

R1-4: The genetic architectures of the included brain disorders are very diverse. The current study design does not seem able to account for the contributions of common versus rare variants, modes of inheritance, levels of polygenicity, etc.

The reviewer is correct that the genetic architectures of the included diseases are quite diverse. The level of analysis necessary to account for rare variants, modes of inheritance, and levels of polygenicity are all important, although we believe less likely to affect major mean profiles and global structure across brain regions. We would like to emphasize that the present study was intended to capture the major transcriptomic and anatomic relationships of disease genes and to compare this with common phenotypic understanding. In the classification presented we have highlighted several associations that are not commonly phenotypically associated and discussed these in the manuscript. We hope the reviewer will see value in presenting these results while acknowledging these identified shortcomings. We have significantly rewritten the manuscript to hopefully better illustrate the value and address caveats and limitations.

R1-5: It would be very helpful if the authors could provide at least some validation of some of their biological predictions. Even if empirical evidence is not possible, some orthogonal form of validation for at least a few of the biological predictions can add confidence to their analysis. For example, do these predictions align with what is known about disease etiology? Where do they disagree? Are there any ground truth data that the authors can benchmark their analyses against?

We thank the reviewer for this comment and the opportunity to look more closely at interpretation. While a major structured validation of the results presented would take us beyond feasible scope,

there are many confirmations of the classification presented here in the literature. Please allow us to illustrate several here. The concurrence of multiple sclerosis and brain tumors has been widely described ((Currie and Urich 1974; Hinnell et al. 2010; Khan, Buwembo, and Li 2005)). A recent study reported that MS patients have a decreased overall cancer risk, but an increased risk for brain tumors (Alkabie et al. 2021). They argue it may be hypothesized that these conditions may occur during the remyelinating processes coinciding with a decline of the CNS immune reaction. Similarly, compared to unaffected controls, patients with brain tumors experienced an independent several fold increased risk of having a prior migraine diagnosis (Chen et al. 2018). Each of these diseases are dominated by expression patterns of ADG1 of **Figure 1**. As an example of the association found in ADG 2, containing most of the neurodegenerative diseases, amyotrophic lateral sclerosis and Alzheimer disease, are seen associated in studies from model systems ((Price et al. 1997) as well as frontotemporal dementia (Ugbode and West 2021). Psychiatric manifestations after occurrence of epilepsy, both diseases of ADG 3, have often been noted. However, the association between newly diagnosed epilepsy and psychiatric disorders afterward is not completely understood ((Ugbode and West 2021; Chang et al. 2013)). Seizures are known to be extremely effective modulators of psychiatric symptoms, and electroconvulsive therapy (ECT) still is used today as one of the most effective antidepressant and antipsychotic treatments. Representative of ADG4, narcolepsy is found associated with excess marijuana use (Bolla et al. 2008, 2010)). Representative of the expression pattern found in ADG 5, Parkinsonian signs of Huntington's disease have been found to typically progress in a fairly linear pattern over time (Reilmann 2019) where bradykinesia is detectable early on in premanifest gene carriers up to two decades prior to the clinical manifestation of Huntington symptoms.

We have rewritten the manuscript to discuss identified associations and included similar evidence for the existence of cortical cell type gradients. While the reviewer may legitimately argue this evidence is anecdotal, and that associations may be found between many disparate disease phenotypes, the literature is consistent with the findings we present. A more startling result which corroborates several of these observations is provided in **Suppl. Fig. 4,** where the mesoscale clustering analysis is presented removing pairwise intersection of gene sets. Surprisingly, the grouping of diseases without gene intersection is highly similar (67% class agreement) to **Figure 1**, suggesting a meaningful transcriptomic association of the diseases. We hope this supplies some evidence for the reviewer.

R1-6: It is important to note that the brain disorders included in this study have very different ages of onset and likely result from pathomechanisms during different times in the lifespan. The current study is performed with adult brain transcriptome data without taking into account developmental expression. This likely confounds the results. For example, genes that cause autism spectrum disorder likely affect prenatal development. The expression of these genes in the adult brain may be very different from the fetal brain and may not be relevant to disease etiology. The absence of temporal expression analysis weakens the study.

The reviewer is of course correct that the diseases presented have very different temporal genetic signatures and thus this is likely to confound associations or even make the proposed association not meaningful. However, we first observe that even genes that likely act mostly in development

to cause pathology may continue to contribute to disease state in adulthood since those genes are still expressed, and neurodevelopmental disorders have symptoms that are persistent across the life span. While we do not claim to capture the developmental aspects of the disorders with our approach, it will provide information about adult pathophysiology. Further, it remains useful, we believe, to elucidate these patterns in adults in comparison with other brain diseases. We have also now examined the presented set of diseases in the BrainSpan (https://www.brainspan.org) data using donors from 60 days old to 39 years. The results highlight the expected temporal patterning and onset of expression in the diseases, while many of the adult associations presented in **Figure 1** remain. We have placed this result in a **Suppl. Fig. 9** and comment on these issues in the main text.

R1-7: The significance of this work is dependent on the strength of the biological insights it provides into these brain disorders. Unfortunately, it is not clear that this work generated deep insights that can form the basis of future studies into disease mechanisms.

We of course agree with the reviewer that a study at this resolution of analysis will not be expected to yield profound results about individual diseases. However, we know of no study where the overall relationship of transcriptomic patterning with respect to the anatomy of the adult brain is presented, and we believe this to be useful and relevant information for those interested in comparing disease relationships in the adult brain. Both expected and less obvious associations are presented as well as identification of those genes responsible and their anatomic presentation. While we do not offer an explicit path toward distinct disease pathomechanisms, we believe the work has value in generating hypotheses that may be followed up through experimental and computational approaches in further studies. Using this work as a model, it will now be possible to improve and generalize the results across far larger data sources that are being developed through BRAIN Initiative work. We have considerably rewritten the manuscript to reflect all of the reviewers essential points and indicate shortcomings of the analysis.

Reviewer #2: Zeighami and collaborators present an integrative transcriptomic analysis of genes associated with 40 common brain diseases representative of 7 phenotypic classes. The authors report that diseases cluster in 5 groups determined by the similarity of expression patterns of their associated genes across anatomical structures of the adult human brain. These expression patterns are reproducible across subjects, generally discriminate among diseases, and only partially relate to phenotypic classes. Comparison with canonical gene expression modules from the Allen human brain atlas further supports distinctions between disease transcriptomic groups and suggest cell type associations underlying some of the differences. To further dissect these associations, the authors analyze expression patterns across cell types from the MTG for 24 diseases with preferential cortical expression. This analysis identified 4 disease groups based on cell type expression patterns and showed that gradients of expression across excitatory and inhibitory neuronal subtypes further distinguish disease groups. Finally, the authors show broad conservation and consistency of cell type enrichment patterns for disease associated genes in both human and mouse, with some exceptions suggestive of species-specific enrichment for a number of diseases and cell types.

We thank the reviewer for this excellent summary which captures the intent for our study.

Overall, their results and approach demonstrate that diseases can be compared and classified based on the neuroanatomic and cell type specific patterns of expression of their associated genes. The study provides an interesting example of how existing brain functional genomics data at different resolutions and in multiple species can be interrogated to better dissect and understand brain disease associations.

As the reviewer comments, our goal was to describe the relationship of neuroanatomic and cell types patterns for major classes of brain diseases, and to characterize their associated disease risk genes. We believe this work provides a valuable overview that is not presented elsewhere and suggests many opportunities for follow up and further analysis, particularly in upcoming large scale human cell atlas work.

The approaches presented are interesting and timely, considering the increasing pace at which gene-disease associations and brain transcriptomic data in human and model species are being mapped. However, I have some concerns regarding the presentation of the results, some unclear methodologies, and the extent of biological interpretation. More clarity and additional biological interpretation complementing data description would largely benefit the study.

These comments on presentation of the results, methodology, and biological interpretation have also been made by others and we clearly hear this critique. We have rewritten the manuscript substantially in an attempt to address these methodological issues, caveats and limitations, and with included increased emphasis on biological interpretation.

R2-1: The style and clarity of the text varies across the manuscript. The syntax and grammar of the first half of the manuscript should be revised. In particular, it is hard to follow the sections: "Introduction", "Brain disorders and associated genes", and "Structural transcriptomic profile of brain diseases". Likewise, the abstract should more closely summarize the data presented and highlight the main contributions.

We have endeavored to address these clear concerns with rewriting of the manuscript. We hope the reviewer will find the manuscript improved.

R2-2: In section "Brain disorders and associated genes": It is not clear why and how the OMIM repository was used. Authors point to reference (14) to support the selection of 549 brain-related diseases to be intersected with the DisGeNET database. Reference 14 does not seem to be related to this. The information included in the associated methods section (Disease genes section) is largely a repetition of what is already included in the main text and does not clarify this issue. There are some inconsistencies between the numbers included in the main text and those included in the methods (e.g., "549 brain-related diseases" vs "an original list of 500 diseases"). Considering that all gene disease association data is coming from DisGeNET, please clarify why

and how the OMIM repository was used. I would suggest clarifying and including most of these details only in the methods section.

We would like to thank the reviewer for pointing out this inconsistency. The reference 14 was in error and should be reference 13: (Hawrylycz et al. 2015). In that study the OMIM database was used to form a list of 549 potential brain-related diseases for a component of that study (We apologize for the inaccuracy, the mismatch between the numbers is now corrected to the correct value of 549.) As DisGeNET contains gene disease associations for many non-brain related diseases, we used the OMIM list as an initial starting point to search for brain related diseases from DIsGeNET, and to be consistent with previous work. We have clarified this in the methods.

R2-3: Diseases are required to have at least 10 associated genes to be included in the study. However, several of the diseases included in Supplementary table 1 contain less than 10 genes. Are all the diseases in Supplementary table 1 included in the study or only a subset? An additional table sheet with descriptions for each data sheet would help clarify this and additional issues regarding the data presented in the tables.

We had included these lower gene diseases as a type of supplementary analysis for several important low gene association diseases (e.g. Opioid-Related Disorders). The analysis originally included ten additional disorders with less than 10 genes based on this prevalence and importance. However due to the potential confusion and since these disorders are not sufficiently discussed in the manuscript, we have now removed this both from the manuscript.

R2-4: In "the proportion of shared genes between diseases is known to be correlated with phenotypic similarity (rho = 0.40, $p = 6.0 \times 10{-}3$)", it is not clear how these numbers were calculated and what they are referring to. How do you measure phenotypic similarity based on the data you have?

This statement is a reference quoted from (Qi et al. 2019). In that study, the authors derived a disease manifestation network (DMN) by curating OMIM and Pubmed. The relationship between the genetic similarity and phenotypic similarity was found correlating phenotype similarity scores from the DMN and shared genes. DMN scores of these disease pairs were found correlated with the proportion of genes shared between diseases at 0.40, $p < 6.0 \times E{-}3$. A similar statistic could be calculated from the results of **Figure 1,** where phenotypic similarity is now correlation between the average expression pattern across major brain structures for a disease pair versus the number of overlapping genes in the pair of disorders and we find (rho = 0.21, $p < 1.0 \times E{-}3$).

R2-5: When listing gene distribution across GBD classes in the format (number, % unique to GBD class), the numbers shown are not percentages.

The percentage is used for Jaccard clustering and is shown as the gray scale color code while the absolute numerical values are written as inset numbers to be consistent with diagonal values,

we have added the text “(shown in gray scale color)” in the **Supp. Fig. 2** legend to further clarify this.

R2-6: In the final disease/disorder list, what is "Dementia" referring to and how is it different from other common causes of dementia also included (e.g. Alzheimer's disease)?

Dementia from DiGeNET is referring to unspecified dementia (ICD10: F03), including dementia with and without behavioral disturbances. The genes reported in “dementia”only include genes reported in studies with patients with unspecified dementia rather than genes associated with dementia with specific underlying neuropathology (e.g. Alzheimer’s disease, ICD10: G30). The information for each disease is detailed in **Suppl. Table 1,** Sheet1 (Brain Disease Attributes), and we have added a new column in **Suppl. Table 1**, sheet 1 with ICD10 codes for each disease to disambiguate these definitions and have referred to this table in the main text.

R2-7: What does structural transcriptomic profile mean?

In the section “Structural transcriptomic profile of brain diseases”, we are discussing the transcriptional pattern of the risk genes for each brain disorder across major neuroanatomy of the brain. To be clearer and more accurate we have changed the title of this section to “Neuroanatomy and the transcriptomic profile of brain diseases.”

R2-8: In Figure 1A, an additional annotation column with the total number of genes for each disease (row) would help with data interpretation. Do diseases in ADG groups 4 and 5 tend to have less genes than diseases in other groups? If so, would that explain the lack of regularities seen in the other, larger ADG groups? An analysis demonstrating that differences in gene number do not play a major role in determining ADG patterns would improve this section.

This is a good point, we have replaced the disease uniqueness annotation bar in **Figure 1A** with an annotation bar indicating gene number. This is likely more relevant to interpreting the results than the more abstract uniqueness score. Most important in reproducibility we believe is the consistent signature across donors. However, the major outliers in gene set size are the neuropsychiatric diseases autistic disorder (g=204), bipolar disorder (g=384), depressive disorder (g=251), and schizophrenia (g=733). We have conducted a reproducibility analysis reducing all gene sets to a maximum of 200 and placed this in **Suppl. Fig.5.**

R2-9: In Figure 1A, it is not clear what uniqueness means.

Uniqueness in this context indicates whether a given transcriptomic profile uniquely identifies the disease in each of the 6 postmortem specimens, that is, by having an expression signature across regions that is most closely correlated with the same disease in other donors. As this is also addressed in panels 1C,D we have replaced this bar with the more informative disease gene number annotation bar.

R2-10 and R2-11:

- Are ADG expression patterns explainable by the degree of gene overlap within classes? It would be interesting to compare the degree of gene overlap (Jaccard index) between diseases of the same ADG group versus the overlap across ADG groups.

- To what extent a small number of "influential" shared genes drives the associations? One way to address this could be by performing a reproducibility analysis similar to those presented in Supp Figures 5 and 6 but this time removing highly pleiotropic (genes) within each ADG group. This analysis would also complement the pairwise analysis presented later in Supp Figure 8. Alternatively, presenting earlier in the text a more extensive description of how the analysis in Supp Figure 8 addresses this problem -- perhaps with specific examples of particularly pleiotropic genes within classes -- would improve the section.

These are interesting and related points. In **Figure 1** all genes associated with each disease are included and so invariably this will be reflected in the ADG groupings. However, we examined the relationship of gene overlap between diseases and ADG membership more closely. A logit regression of common ADG membership against normalized gene set intersection (intersection/union) finds highly significant coefficients (intercept $p<2e-16$, overlap $p<3.58e-11$). However, normalized gene set intersection for within ADG pairs is 0.029 compared with 0.016 for non-ADG pairs, and while a one-sided t-test shows this is significant ($p<4.8e-09$) the difference in magnitude is not large. The reviewer is correct that the results could be regenerated removing influential and highly pleiotropic genes, however, the result of **Supp. Fig. 4** is essential to this point and shows that ADG membership is reproducible by comparing common pairs of diseases with and without shared genes agrees at 67% which is quite strong.

R2-12: Figure legend explanations for panels C and D in Figure 1 are not very clear. Axes labels are missing. This analysis is very interesting, but the results are hard to follow as currently presented in the main text and figure. Are the authors trying to show that the anatomic pattern of a given disease in one subject tends to be similar to patterns in another subject for the same disease or disease of the same class -- and not to patterns of different diseases?

We apologize for this lack of clarity, and realize the explanation given was imperfect. The goal of the analysis is to examine the transcriptomic pattern of a disease for one of six donors compared with other donors with respect to their ADG and GBD classification. This analysis is a study in the variability and reproducibility in the patterns across donors. In this way we consider the anatomic transcriptomic profile for each disease in a given donor and ask which profile is it most closely correlated with in other donors. The result may be that it is highest correlated with the same disease in another individual, with another disease in the same ADG, or with some other disease. This forms a measure of the uniqueness of disease profiles and the stability of ADG groups across donors. For example in 1C, diseases of ADG4 in one donor are always most closely correlated with ADG4 in other individuals, and almost always identifies (0.96) the exact disease. The stability

of ADG membership in this way is quite strong, and in the majority of cases the exact disease can be identified in another donor. We believe the unique identifiability of a disease through its anatomic transcriptomic signature is an interesting and important concept that may be useful in distinguishing and further characterizing diseases. With respect to phenotypic GBD classes, movement and substance abuse disorders are seen to have the most reproducible and unique profiles, while psychiatric and developmental the most variable across donors. We have enriched this explanation in the text and also added the labels for the corresponding axis.

R2-13: This statement is not clear: "The ability to uniquely identify a disease from its anatomic signature indicates a finer transcriptomic patterning and is a bridge to cell type analysis".

The intent of our statement is that the extent to which a disease can be identified by its transcriptomic signature across neuroanatomy is also indicative of specialization of the cell types expressed by genes associated with that disease. A more unique and reproducible transcriptomic signature across anatomical structures for a given disease may provide indication of an approach to cell type characterization of that disease. We have now modified the original text to clarify this point.

R2-14: Have the authors considered whether the fact that different diseases show different degrees of cross-subject anatomical profile similarity could relate to their underlying neurobiology? For example, given their developmental origin and high phenotypic heterogeneity, is it expected that psychiatric and developmental diseases show the least consistency? Some level of discussion of this would be interesting.

This is an interesting point which is to a certain extent summarized in **Figure 1D** and **Supp. Fig. 11-B,C**. We have discussed **Fig 1D** above which measures between subject variability of anatomic profiles by GBD class. **Suppl. Fig. 11** uses the concept of differential stability (DS) proposed in (Hawrylycz et al. 2015) as a measure of the transcriptomic consistency of *individual genes* across donors, based on preserved differential relations between anatomic structures, and provides a complementary perspective. Whereas in terms of cross-subject consistency in **Fig 1D** substance abuse and movement disorders are highest, and developmental and psychiatric diseases least, when examining the stability of individual disease genes by donor, we find that while substance abuse diseases have the most stable gene patterns, and tumor and neurodegenerative diseases the least.

The differential stability measure prioritizes neuronal cell types with strong structural markers and less so the non-neuronal broad non-regional expression common in glial cells. While gene expression variability may be potentially related to higher heterogeneity within a disease, consistent anatomic patterning may suggest shared underlying cellular pathways between these disorders and a spectrum with both shared and unique phenotypic elements. Another method of examining this is shown in the module analysis of **Figure 2** of the manuscript. Here genes for each disease that are associated with each of M1-M31 canonical expression patterns are

presented, with higher values (deeper red) indicating a higher fraction of genes associated with that module. The modules are ordered by decreasing neuronal cell type content, as shown in the upper annotation bar. **Figure 2** shows the over-representation of neuronal markers in diseases of movement and substance abuse and glial patterning for ADG 1 and ADG 2.

R2-15: Brain disorders are classically defined based on observable neuropathological signatures (e.g., degenerative disorders) and/or behavioral symptoms (e.g. psychiatric disorders). There has been much discussion in the field regarding intrinsic limitations when trying to understand the neurobiology linking genes to brain disease phenotypes. Because multiple levels of organization are involved (molecular, cellular, circuit, behavioral, etc…), it is not clear whether certain accessible endophenotypic levels might be more appropriate than others to study disease.

- Have the authors considered interpreting/discussing some of their results in such a context? For example, some disease phenotypic classes show more consistent transcription patterns than others, and some diseases are more transcriptionally similar to diseases in other classes. Does this suggest that phenotype classes might not capture the relevant underlying neurobiology and need revision, or that molecular level endophenotypes are not equally informative across brain disease classes? Some level of discussion of these aspects would improve the representation of your results.

We thank the reviewer for the observation that some disease phenotypic classes show more consistent transcription patterns than others, and some diseases are more transcriptionally similar to diseases in other classes. It is one of our primary results and motivations of this work to suggest that common phenotype classes based on disease manifestation and presentation may not fully capture underlying neurobiology and allow for revision. It is also certainly the case that molecular level endophenotypes are not equally informative across brain disease classes as seen in variability results. We have now added text in the discussion to further emphasize this motivation and the implications of the clustering analysis.

R2-16: Regarding the use of existing canonical modules to aid interpretation, the following statement is very interesting, and perhaps could be expanded in the discussion section: "Brain wide association of expression module profiles may potentially implicate genes without previous association to a given disease, particularly when that profile is highly conserved between donors".

Thank you for this observation, we have expanded this application in the discussion.

R2-17: In "Averaging τ over sets of genes representing a given disease, we obtain a measure of cell type specificity of each disease within MTG (Suppl. Fig 14C)", figure reference seems incorrect.

We have now corrected the reference.

R2-18: The analysis in Figure 4 A and B is not very clear and the associated methods are very sparse. The axes are not labeled. Columns and rows seem to be cell types. What profiles are being used to compute covariation? What does cell type interaction mean in this context? How can single disease and disease-pair entries be defined based on this analysis? How do you go from this analysis to the genes in Figure 4B?

We apologize for the adequate treatment of **Figure 4** and have modified and improved the content and presentation to more fully describe our intended results. In particular the methods described were inadequate and led to confusion. Please see the main text indicating clarification of this description. In particular, In **Figure 4A** gene expression covariation is computed as the absolute value of cosine distance similarity of cell type expression across MTG cell types. matrices are computed for each of three psychiatric diseases (Aut, Bip, Scz), and then independently thresholded to 1.5σ. These matrices represent a measurement of potential cell type interaction between MTG cell types for a given disease. Entries are combined into a single matrix and are color coded if a given disease exceeds the threshold. **Figure 4B** shows detail of the excitatory cell types. The legend indicates individual, pairs, or all three diseases showing positive interaction.

R2-19: In Figure 5A, in the interspecies cellular taxonomy, it is not clear what the squares in the bottom represent. Is it the number of matches? Additional labels and more description would help.

We have now added the labels for the figure and in the description. The squares in **Figure 5A** represent the number of clusters in the mouse or human taxonomy that are mapped to the shown homologous consensus cluster. We have clarified this in the text.

R2-20: In Figure 5B, it is not clear what scores are being shown. What type of scores EWCE analysis uses? Figure 5B shows positive numbers close to 1.0, while Figure 5C shows positive and negative numbers. Does the permutation analysis use z-scores to quantify enrichment? Are the values in Figure 5B -log(p-values)? If so, the fact that most values are close to 1.0 indicates that disease gene expression patterns are not cell type specific (enriched) similarly in human and mouse? Please clarify and add figure labels.

We apologize for the lack of clarity in this presentation, and we have improved this figure. We have modified the figure to better highlight the intended results. **Figure 5C** now presents the cell type enrichment EWCE values co-clustered for mouse and human. Figure 5D presentes the co-clustered expression values, highlighting the interesting species specific differences. The original panel which appeared in this figure is now part of **Suppl. Fig. 20.** This panel shows the significant corrected p-values in both species. This is now clarified in the main text.

EWCE compares the expression levels of the genes associated with a given disease to the background gene expression (all genes, excluding the disease-related genes) by performing permutation analysis and defining the probability for the observed expression level of the given gene set compared against a random set of genes. **Figure 5B** shows the distribution of EWCE values of all diseases and cell types in mouse and human in the consensus taxonomy of **Figure**

5A. These values are calculated in a given cell type for each of the 40 disorders based on the genes involved and the histograms presents these values across all 800 cell-disease pairs (40*20=800) in mouse and human dataset. Therefore an entry in the histogram is the EWCE value for a given disease in a given consensus cell type. A value of 1.0 indicates neither enrichment or deficit and so it is logical that this distribution would be centered. We then used a Two-sample Kolmogorov-Smirnov test to compare them showing only marginal differences.

R2-21: The abstract mentions that comparisons with mice somehow indicate "where human data is needed to further refine our understanding of disease-associated genes". However, there is no data related to this point in the manuscript.

We apologize for indicating that this data was used in the manuscript. We have changed this to "will be needed to further refine our understanding of disease associated genes."

R2-21: I would suggest revising the title, in particular the word foundations does not provide any information.

This is a good suggestion. We have modified the title of the manuscript to "Anatomic and cellular transcriptome structure of human brain disease."

Reviewer #3: This paper claims that 40 common brain diseases can be aggregated into 5 groups according to the anatomical expression of the genes associated with these brain diseases in the adult brain.

In this manuscript, gene sets for 40 brain diseases are collated from DisGenNET database, then the expression of these gene sets is examined in adult RNA-seq data from the Allen Human Brain Atlas across 104 structures and single-nucleus data for 75 cell types from the medial temporal gyrus. Gene expression data were averaged across gene sets for each disease and brain structure, then the gene expression data were clustered to define 5 Anatomic Disease Groups. Mean differences across groups and each structure were then calculated to quantify expression differences, so Mean of Mean analysis (a statistician should evaluate the merits of this analysis). By investigating the co-expression modules initially reported for the Allen brain dataset, distinct modules are noted for brain regions. Brain disorder genes with high cerebral cortex expression were then examined for cell type enrichment in the MTG dataset. They also compare to mouse cell types.

We thank the reviewer for this summary. We would like to reiterate that the overarching goal of this study was to investigate and describe the large-scale anatomic and cell type patterning of disease risk-associated genes in the brain. It is clear that any attempt to summarize profiles and differences at this level of organization will necessitate an average analysis which will of course obscure the finer molecular structure. We would like to reiterate that we analyze expression patterns for risk genes in post mortem brains without, for example, neuropsychiatric illness and that we can not infer differential gene expression in brains of patient populations.. The primary results presented in **Figure 1** and **Figure 3** use an average expression profile and comparison across anatomic structures will result in comparing differences of means. This type of comparison is not uncommon in statistics, such as in the use of an omnibus statistic where a statistically significant significant difference is observed but the test does not specify exactly where the difference occurred, or specify which parameter is significantly different from another, but determines that there is a difference. We agree that while this approach is unable to identify disease-specific gene expression changes, the novelty here is precisely in describing brain-wide anatomic and transcriptomic structure across a broad class of diseases, which we do not believe has been elucidated elsewhere.

R3-1: The authors pose the following hypothesis: spatial and temporal co-expression of disease genes is indicative of a potential interaction between genes associated with brain diseases. Since the RNA datasets included in the analysis are derived from samples of adult brains, they provide no context for temporal relationship with gene expression. Similarly, co-expression is not a proxy for interaction, as RNA-protein are often not expressed at the same time or in the same cells (e.g., PMID: 35288716).

This critique was appropriately raised by another reviewer and is of course correct that the diseases presented have very different temporal genetic signatures and thus this is likely to confound associations or even make the proposed association not meaningful. However, we observe that even genes that likely act mostly in development causing pathology may continue to

contribute to disease state in adulthood since those genes are still expressed. While we do not claim to capture particularly the developmental aspects of the disorders with our approach, it will still provide information about adult pathophysiology. Further, it remains useful, we believe, to elucidate these patterns in adults in comparison with other brain diseases. We have examined the presented set of diseases in the BrainSpan (https://www.brainspan.org) data using donors from 60 days old to 39 years. The results highlight the expected temporal patterning and onset of expression in many of the diseases, while many of the adult associations presented in **Figure 1** remain. We have placed this analysis in **Suppl. Fig. 7** and comment on these issues in the main text.

R3-2: Thus, the data do not address the stated hypothesis. Rather, the data reflect the following question: Do various brain diseases aggregate based on the anatomical location of associated gene expression in the adult brain? The authors should address these limitations in their manuscript and edit their stated hypothesis, or include data (e.g. BrainSpain) that would allow for temporal analysis. In this context, there are many references to the patterning of the cortex in the results and discussion sections which seems to refer to the enrichment of gene expression patterns in the cortex which is different from enrichment in neuronal patterning, which is a developmental process. Thus the complete manuscript should be reviewed and edited for clarity.

We thank the reviewer for this observation and agree that the primary goal of this work is addressing the question of whether brain disease exhibits similarities based on anatomic location of associated gene expression in the adult brain. We thought we had been clear that this was the intended goal and have endeavored to revise the manuscript to present this more clearly. We apologize for having confounded enrichment in neuronal patterning with enrichment of expression patterns and have revised the manuscript in an attempt to make clear that we are not able to make concrete statements about developmental patterning, beyond basic inclusion of the BrainSpan analysis.

R3-3: Among the diseases included in the analyses, several are known to impact an overlapping set of brain structures - it would be helpful if the results were placed into this context of the known structures that are impacted. As already noted, many of the diseases investigated have known origins in early brain development, which is not addressed/discussed.

We appreciate this comment and agree it would be informative to compare the present results with known brain structure involvement. We investigated the possibility of including this analysis and conducted a literature survey to tabulate regional brain involvement in diseases based on the major brain structures used in **Figure 1,** cortex, hippocampus, amygdala**,** etc. The results however indicate literature reference to essentially any major structure of the brain in connection with a given brain disease. This made highlighting comparison with transcriptomic structural profiles difficult. The results for MTG cell type specific data are new and their is insufficient literature reference. We agree that a systemic analysis would be valuable and would like to investigate this in a separate study.

R3-4: Cerebrovascular diseases, despite being the most burdensome brain diseases, were excluded from analysis due to the limited representation of relevant cell types in the datasets utilized. It would be helpful to provide a power calculation of the sample size needed for the RNA-seq datasets to be suitable for analysis. Despite this stated limitation, the non-neuronal MTG cell types were included in analysis for those brain diseases that were retained for analysis. More generally, it would be helpful to calculate the power for each analysis since the data and gene expression vary by region (number of genes expressed per region). Similarly, if the authors deconvolute the bulk cortex samples based on the MTG data, do they achieve similar results?

This is a good point although potentially very challenging to implement in practice. The analyses employed are identifying statistically significant differences and which evidently take into account sample number, mean values and variances. With respect to the MTG cell type data, identification of cell types and minimum number of cells to identify types of given frequency of occurrence was accomplished in the reference Hodge et al, 2019 which the present work uses. This power calculation was achieved using the tool https://satijalab.org/howmanycells/ and we are confident that the observed types are valid based on that work.

R3-5: In the discussion, the authors should describe the novelty and implications of their results, which is not clearly described in the current version. Overall, the current manuscript requires significant revision for clarity and context to be suitable for publication.

We appreciate the reviewer's perspective that the writing in the manuscript, while attempting to be technically precise, does not adequately describe the novelty and implications of the results. We have substantially revised the manuscript with the reviewer's comments in mind and hope this new presentation is more convincing and appropriate.

References

Alkabie, Samir, Rhaisa Castrodad-Molina, Kent A. Heck, Jacob Mandel, and George J. Hutton. 2021. "The Concurrence of Multiple Sclerosis and Glioblastoma." *Multiple Sclerosis and Related Disorders* 50 (May): 102877.

Bolla, Karen I., Suzanne R. Lesage, Charlene E. Gamaldo, David N. Neubauer, Frank R. Funderburk, Jean Lud Cadet, Paula M. David, Antonio Verdejo-Garcia, and Amy R. Benbrook. 2008. "Sleep Disturbance in Heavy Marijuana Users." *Sleep* 31 (6): 901–8.

Bolla, Karen I., Suzanne R. Lesage, Charlene E. Gamaldo, David N. Neubauer, Nae-Yuh Wang, Frank R. Funderburk, Richard P. Allen, Paula M. David, and Jean Lud Cadet. 2010. "Polysomnogram Changes in Marijuana Users Who Report Sleep Disturbances during Prior Abstinence." *Sleep Medicine* 11 (9): 882–89.

Chang, Hsiu-Ju, Chien-Chang Liao, Chaur-Jong Hu, Winston W. Shen, and Ta-Liang Chen. 2013. "Psychiatric Disorders after Epilepsy Diagnosis: A Population-Based Retrospective Cohort Study." *PloS One* 8 (4): e59999.

Chen, Chao-Hung, Jau-Jiuan Sheu, Yi-Chun Lin, and Herng-Ching Lin. 2018. "Association of

Migraines with Brain Tumors: A Nationwide Population-Based Study." *The Journal of Headache and Pain* 19 (1): 111.

Currie, S., and H. Urich. 1974. "Concurrence of Multiple Sclerosis and Glioma." *Journal of Neurology, Neurosurgery, and Psychiatry* 37 (5): 598–605.

Hinnell, C., M. Almekhlafi, J. T. Joseph, R. Bell, P. Sharma, and S. Furtado. 2010. "Concurrence of High-Grade Brainstem Glioma and Multiple Sclerosis." *The Canadian Journal of Neurological Sciences. Le Journal Canadien Des Sciences Neurologiques* 37 (4): 512–14.

Khan, Shahnaz Hayat, Joseph E. Buwembo, and Q. Li. 2005. "Concurrence of Glioma and Multiple Sclerosis." *The Canadian Journal of Neurological Sciences. Le Journal Canadien Des Sciences Neurologiques* 32 (3): 349–51.

Price, D. L., P. C. Wong, D. R. Borchelt, C. A. Pardo, G. Thinakaran, A. P. Doan, M. K. Lee, L. J. Martin, and S. S. Sisodia. 1997. "Amyotrophic Lateral Sclerosis and Alzheimer Disease. Lessons from Model Systems." *Revue Neurologique* 153 (8-9): 484–95.

Reilmann, Ralf. 2019. "Parkinsonism in Huntington's Disease." *International Review of Neurobiology* 149 (November): 299–306.

Ugbode, Chris, and Ryan J. H. West. 2021. "Lessons Learned from CHMP2B, Implications for Frontotemporal Dementia and Amyotrophic Lateral Sclerosis." *Neurobiology of Disease*. https://doi.org/10.1016/j.nbd.2020.105144.
