# Supplementary material for: A comparison of anatomic and cellular transcriptome structures across 40 human brain diseases
Source: PLoS Biol. 2023 Apr 20;21(4):e3002058. doi: 10.1371/journal.pbio.3002058 (PMC10118126; doi:10.1371/journal.pbio.3002058)
Supplement: Attachment — Submitted filename: Reviewer_Response_Final.docx [file pbio.3002058.s011.docx]

**Reviewer #1: In this revised manuscript, Zeighami et al acknowledged many of the weaknesses pointed out by the reviewers in the last round of reviews. The revisions however were largely textual; the limitations, although now formally acknowledged, remain unresolved and still impact the analyses and the significance of the overall study.**

Previously, I pointed out that the success of the authors' strategy hinges on gene selection for each of these complex and very different brain disorders. The problems are 1) the disease burden carried by each gene can vary significantly (some carry significant burden whereas others are risk factors), 2) the strength of the evidence supporting each gene also varies a great deal (some are convergently supported by multiple large cohort studies whereas others have conflicting data), 3) the nature of the mutations causing each disease (i.e. loss-of-function, gain-of-function, neomorphic, regulatory) or the mode of inheritance were also not considered. In the revised manuscript, the authors used the gene-disease association (GDA) score from DisGeNET, which considers the number and type of sources, and the number of publications supporting gene-disease association and presented the data in Suppl. Fig. 8. Although this analysis addressed point 2 above (on the strength of the evidence), point 1 (on disease burden/effect size) and point 3 (on the type of mutation or mode of inheritance) remain important issues that have not been resolved, limiting the strength of the analyses and the significance of the study.

We appreciate the reviewer’s attention to these key details, and the reviewer is of course correct that accurate gene selection is critical to precise identification of a disease and the understanding of its anatomic profiles and phenotypic significance. Disease burden will vary significantly depending on the factors mentioned above, however the annotation proposed while quite valid is evidently neither available nor fully understood by the community. To fully complete the analysis proposed accounting for all these criteria would require a profound and extensive annotation of the existing literature and databases and would undoubtedly require further vetting by multiple experts, who would likely have disagreement about effect size and other issues. We are pleased however that the reviewer acknowledges addressing the strength of evidence through the inclusion of the new Suppl. Fig. 8. We acknowledge that disease burden, effect size, type of mutation, and mode of inheritance remain not accounted for by this analysis and factors cannot be fully identified through the DisGeNET associations. In the present work, we have approximated a solution to the identification of disease risk, allowing us to describe the anatomic relationships of general characteristics of these diseases. We believe furthermore that it is unlikely that further depth in annotating gene sets would substantially change the results presented.

In the first round of reviews, another reviewer and I each raised the point that the brain disorders included in this study have very different ages of onset and likely result from pathomechanisms during different times in the lifespan. The use of adult brain transcriptome data without considering developmental expression likely confounds the results. The authors responded that they "observe that even genes that likely act mostly in development to cause pathology may continue to contribute to disease state in adulthood since those genes are still expressed, and neurodevelopmental disorders have symptoms that are persistent across the life span." What is the evidence supporting this statement? Genes that are required for brain development can be expressed in adult but play a much different or less functional role. They can also shift in their expression patterns from development to adulthood. Persistent adult symptoms from neurodevelopmental disorders can originate from altered developmental processes that have a lasting impact through life that have nothing to do with the adult expression of the gene. I do not understand the logic of the authors' argument here.

We would like to thank the reviewer for giving us a chance to clarify our hypothesis. First, we certainly agree with the reviewer that gene expression patterns in adulthood obviously do not necessarily reflect developmental expression. Hence, we would not claim that our findings in adult brains directly relate to developmental processes. However, we do regard it as potentially salient findings that risk genes for neurodevelopmental disorders show co-expression also in adult stages. While we certainly also agree that persistent adult symptoms can originate from altered development, which has a lasting impact through lifespan, we would argue that co-expression of risk genes mapping to distinct biological pathways in adulthood might result in disruptions of these respective processes, potentially contributing to the persistence of symptoms. Further, the developmental onset of diseases is highly variable, and perhaps the only way to robustly include this axis would be through a normalized pseudo-time analysis, with the ability to compare disparate time trajectories, and likely requiring a far larger data set for the analysis. As our approach does not allow for experimental manipulations to allow causal inference, we have made clear now that this assumption is a hypothesis that needs subsequent testing.

Furthermore, the authors "have also now examined the presented set of diseases in the BrainSpan ([https://www.brainspan.org](https://nam12.safelinks.protection.outlook.com/?url=https%3A%2F%2Fwww.brainspan.org%2F&data=05%7C01%7C%7Cad6dff4b083c45f48f0c08db1034b2a7%7C32669cd6737f4b398bddd6951120d3fc%7C0%7C0%7C638121590769758816%7CUnknown%7CTWFpbGZsb3d8eyJWIjoiMC4wLjAwMDAiLCJQIjoiV2luMzIiLCJBTiI6Ik1haWwiLCJXVCI6Mn0%3D%7C3000%7C%7C%7C&sdata=c%2FdGQhuxcdgSwxKw8VQy%2BP0%2FpwhXu4OZ2TdnKHzs9Do%3D&reserved=0)) data using donors from 60 days old to 39 years. The results highlight the expected temporal patterning and onset of expression in the diseases, while many of the adult associations presented in Figure 1 remain. We have placed this result in a Suppl. Fig. 9 and comment on these issues in the main text." I do not see what in Suppl. Fig. 9 supports the assertion that "many of the adult associations presented in Figure 1 remain"; 27/40 disorders now fall under a single large group; the other groups are a mix of diseases from previously disparate groups. Importantly, disorders with very different ages of onset, for example Autism and Alzheimer, fall under the same group. There are clear discrepancies compared to the original analysis.

The clustering presented is a summary over the 13 developmental time points from 60 days to 39 years old and presents the average expression of associated genes for the 40 diseases. The groups reflected by the clustering are naturally presented at a comparatively high level. While 27 of the diseases fall into a single group, more importantly, within that group the psychiatric disease, substance abuse, and neurodegenerative diseases are grouped proximally as they are in the adult. Further, the diseases more closely associated with white matter expression of Anatomic Disease Group 1 (ADG 1) from Figure 1 of the manuscript are similarly grouped. The developmental trajectory of expression shows that the adult signature of Figure 1 in the manuscript is consistent with many of the diseases. We reiterate that this developmental analysis is coarse and intended only to describe the general time trajectory.

An important issue that I previously brought up is that the significance of this work is dependent on the strength of the biological insights it provides into these brain disorders. Unfortunately, it is not clear that this work generated deep insights that can form the basis of future studies into disease mechanisms. The authors, "of course agree with the reviewer that a study at this resolution of analysis will not be expected to yield profound results about individual diseases." If this is the case, then what is the substantive contribution of this study overall?

While we appreciate the reviewer’s point, as we have indicated, we believe the value in this study is in the overall description of the anatomic gene expression relationships between these important diseases. The analysis provided will not be expected to indicate a clear path to the understanding of the specific neuropathology of individual diseases at a deep molecular level. Rather, we believe the brain wide description of these diseases and their neuroanatomic patterning, as well as the relationship to cell type specific expression in the cortex provides a novel way of aggregating and classifying diseases that has not been previously described. We believe this work provides researchers as well as students of the field with a previously underappreciated view of large-scale molecular relationships between major disease classes. The analysis provides a resource guide to genetic disease associations that will be important for consideration in follow up experiments and in associating phenotypic relationships.

In the present form, this work represents a set of predicted relationships between disorders that have not been orthogonally validated. In addition to the validity of the predictions, the real-world utility of the work is also in question. The authors state that they "believe the work has value in generating hypotheses that may be followed up through experimental and computational approaches in further studies." However, I did not find where the authors described concrete, testable hypotheses generated based on their analyses. The authors argue that their study "is a step toward a biologically driven approach that uses transcriptomic and cell/pathway data to inform brain disorder classification." However, it does not seem to inform the clinical classification, diagnosis, or treatment strategies of these disorders. The real-world impact of the work is therefore not clear. A major take home seems to be that "we observe that disease risk genes show convergent physiological based expression patterns that associate diseases in expected and sometimes less expected ways." This speaks neither to the validity nor the utility of the predictions.

We appreciate the reviewer’s concerns here, and while we have not provided follow up experiments externally validating the results presented, a sizeable survey of the literature confirming the plausibility of many of the associations is described. While we have not indicated treatment strategies for the diseases, an important premise of the work is offering a novel way of clinically classifying diseases based on transcriptomic anatomic patterning. We believe one would not expect a work of this nature to reliably indicate diagnosis strategies.

**Reviewer #2: The authors have addressed all my previous comments and suggestions. The updated manuscript is much improved.**

We thank the reviewer for acknowledging the improvements made in the manuscript and are grateful for the reviewer’s insights.

As final comments:

I suggest further revising the abstract to delineate more clearly what constitutes background knowledge, what are the analytical contributions of the current study, and what are the major findings.

This is an important clarifying observation, and we have revised the abstract to reflect the background, new contributions, and major findings of the study more clearly.

I suggest adding a description sheet to each Supplementary table to clearly describe the different data and features being presented.

We have added a description of each Supplementary Table explaining the contents and features of each.

**Reviewer #3: The authors have thoughtfully and appropriately responded to the reviewers' comments. The substantial revisions in the current manuscript have clarified the results and interpretations and include important limitations.**

We thank the reviewer for acknowledging the improvements made in the manuscript and are grateful for the reviewer’s insights.
